# Long-Term Physical Activity Participation and Subsequent Incident Type 2 Diabetes Mellitus: A Population-Based Cohort Study

**DOI:** 10.3389/fendo.2021.769549

**Published:** 2021-11-30

**Authors:** Chenglong Li, Yanjun Ma, Rong Hua, Fanfan Zheng, Wuxiang Xie

**Affiliations:** ^1^ Peking University Clinical Research Institute, Peking University First Hospital, Beijing, China; ^2^ Peking University Clinical Research Institute Heart and Vascular Health Research Center at Peking University Shougang Hospital, Beijing, China; ^3^ Key Laboratory of Molecular Cardiovascular Sciences (Peking University), Ministry of Education, Beijing, China; ^4^ School of Nursing, Peking Union Medical College, Chinese Academy of Medical Sciences, Beijing, China

**Keywords:** prediction and prevention of type 2 diabetes, clinical science, epidemiology, exercise, obesity

## Abstract

**Background:**

Uncertainty remains concerning association between long-term physical activity and incident type 2 diabetes mellitus (DM). We intended to evaluate physical activity participation over a 6-year span and assess association with subsequent 10-year incident DM risk, as well as examine mediation role by obesity.

**Methods:**

A total of 9757 community-dwelling adults aged ≥ 50 years in England were included in the population-based cohort. Physical activity participation, including trajectories and cumulative participation were assessed using weighted ***Z*** score over a 6-year span from wave 1 (2002–2003) to wave 4 (2008–2009). Incident DM recorded over a 10-year span from wave 4 (2008–2009) to wave 9 (2018–2019) was outcome.

**Results:**

5 distinct activity trajectories were identified, including persistently low (N=3037, incident DM=282), initially low then improving (1868, 90), initially high then declining (325, 20), persistently moderate (2489, 170), and persistently high (2038, 108). Compared with persistently low, participants of initially low then improving, persistently moderate and high were associated with lower incident DM risk, with multivariable-adjusted hazard ratios (HR) of 0.41 (95% confidence interval [CI]: 0.32 to 0.53, *P*<0.001), 0.70 (95% CI: 0.56 to 0.89, *P*=0.004) and 0.49 (95% CI: 0.37 to 0.65, *P <*0.001), respectively. Elevated cumulative activity was also associated with lower DM risk, with each quintile increment in cumulative weighted ***Z*** score corresponding to HR of 0.76 (95% CI: 0.71 to 0.82, *P <*0.001). Mediation analysis found that body mass index, waist circumference and change in body mass index mediate 10% (*P <*0.001), 17% (*P <*0.001) and 9% (*P <*0.001) of the observed association between activity and incident DM, respectively.

**Conclusions:**

For middle aged and older adults, both gradually improved and persistently active participation in physical activity were associated with subsequent lower risk of incident DM, with obesity playing a potential mediator. Strategies focusing on improving and maintaining active participation in physical activity might be beneficial from DM prevention perspective.

## Introduction

According to data from the Global Burden of Disease, the global annual incident type 2 diabetes mellitus (DM) has exceeded 20 million in the year 2017, increased by 30.5% compared with the year 2007 (1). The key role by physical activity in the prevention of DM has been well-established by previous evidence, from both interventional and observational studies (2–8). However, considering that physical activity could experience considerable alterations in long-term and the relatively low incidence of DM, longer period was needed for both comprehensive physical activity evaluation and assessment of its association with subsequent DM risk reduction. So evidence from interventional studies could not evaluate the long-term trajectories of physical activity and its long-term impact on DM risk reduction, due to limited follow-up period (3–6). And most observational studies only evaluated physical activity at single timepoint of study entry, without accounting for the longitudinal change and cumulative activity participation in a long-term period (7, 8). More importantly, few studies have incorporated the role of obesity into consideration when evaluating the association between physical activity and incident DM. As reported by previous studies, the association between changes in physical activity and incident DM could be attenuated, when further adjusting for obesity in follow-up (5, 9). So it was possible that obesity could play as a mediator between physical activity and incident DM, which remained an unanswered question.

Therefore, we intended to investigate the long-term physical activity participation during a 6-year span based on a life-span approach, by evaluating activity trajectories and cumulative participation, and assess its association with subsequent 10-year risk of incident DM among community-dwelling adults aged ≥ 50 years. We also aimed at exploring whether obesity, assessed by body mass index (BMI) and waist circumference, played the mediating role between long-term physical activity and incident DM.

## Materials and Methods

### Study Population

The English Longitudinal Study of Ageing (ELSA) is an ongoing prospective and nationally representative cohort of community-dwelling adults aged ≥ 50 years in England. Details concerning objective, design, and method of the study have been published (10). The ELSA was approved by the London Multicentre Research Ethics Committee (MREC/01/2/91), with informed consent obtained for all participants. We used survey data from waves 1 (2002–2003) to 4 (2008–2009) to evaluate physical activity participation, and waves 4 (2008–2009) to 9 (2018–2019) to assess incident DM. Participants were excluded if they had been diagnosed of DM by wave 4. Study timeline was presented in **Supplemental Figure 1**.

### Physical Activity Assessment

Biennial evaluation of frequency in participating mild, moderate and vigorous intensity physical activities was performed using three questions, differentiating from work related activities. Answers included 4 categories: 1) hardly ever or never; 2) one to three times per month; 3) once per week; 4) more than once per week. Further details were presented in **Supplemental Methods**.

A three-stage approach was used to assess physical activity participation. Firstly, we assigned a score of 1, 2, 3 according to frequency of participating in activities of mild, moderate and vigorous intensity, respectively. A score of 1 was assigned for hardly ever or never, 2 assigned for one to three times per month, 3 assigned for at least once per week. Score assignment was the same for the three types of activity (11). Secondly, for each of three types of activity, we calculated standardized ***Z*** score by subtracting corresponding mean and dividing by standard deviation (SD) of assigned scores at baseline. An activity ***Z*** score of 1 at a certain wave indicated that the particular score of physical activity at the wave was 1 SD higher than the average score of physical activity at baseline. Similar approach can be found for handling cognitive test scores by previous studies (12–14). Thirdly, to account for differences between mild, moderate and vigorous intensity activities, a weighted global activity ***Z*** score was summarized. The weights were selected based on metabolic equivalent of tasks (MET) estimates by 2011 Compendium of Physical Activities (15). After calculation, MET weights of 2.3, 4.4, and 7.2 were assigned to mild, moderate, and vigorous intensity activities ***Z*** score, respectively. Detailed information for weights calculation was presented in **Supplemental Table 1**, which was consistent with previous studies (16).

### Obesity Assessment

The ELSA performed physical measurement at regular intervals, with standardized protocols applied, as described in **Supplemental Methods**. Measurement was conducted by trained nurses for all participants in wave 0, 2, 4, 6, 8 (the year 1998, 2004, 2008, 2012, 2016). We used data in wave 4 to assess obesity, including BMI and waist circumference.

### Ascertainment of DM and Prediabetes

According to the 2014 American Diabetes Association guidelines (ADA), DM was defined as an fasting plasma glucose ≥ 126 mg/dL (7.0 mmol/L) or an HbA1c level ≥ 48 mmol/mol (6.5%), a self-reported physician diagnosis of DM or current use of glucose-lowering therapy (17–19). Among participants without DM, we defined prediabetes as an fasting plasma glucose in the range 100 mg/dL (5.6 mmol/L) to 125 mg/dL (6.9 mmol/L) or an HbA1c level in the range 38.8 to 46.4 mmol/mol (5.7 to 6.4%) (17). The ELSA also collected information about the age being newly diagnosed of DM by physicians since last visit. Time to incident DM was calculated as length between age at wave 4 and age of firstly being diagnosed.

### Covariates

Covariates included demographic and clinical variables assessed at wave 1. Demographic variables included sex, age (years), ethnicity, educational background (high-level education or not), cohabitation status (living alone or not), current smoking (yes or no), alcohol consumption (at least once per week). Clinical variables included overweight status, depressive symptoms, hypertension, prediabetes status, stroke, cardiovascular diseases, cancer, and chronic lung diseases. Considering that elder participants could experience decline in physical activity participation due to deteriorated mobility, we further adjusted for the mobility status.

We defined high-level education as above senior level of high school or 12 or more years of education. Mobility status was defined as whether participants reported any difficulties in daily living activities during waves 1 to 4. These activities included eating, bathing, dressing, getting in/out of bed and walking across a room, which could reflect the general mobility status. Depressive symptoms were defined based on an 8-item version of the Center for Epidemiologic Studies Depression Scale (CESD-8), with scores ≥4 regarded as having depressive symptoms (13, 20). Overweight status was defined by BMI (≥25 or <25 kg/m^2^) according to World Health Organization (21). Personal history of other diseases was derived from records of self-reported physician diagnoses.

### Statistical Analysis

For characteristics description, mean ± SD was used for continuous variables and numbers (percentage) for categorical variables. Overall differences between groups were tested using analysis of variance (ANOVA) or chi-square test.

**Figure 1:**
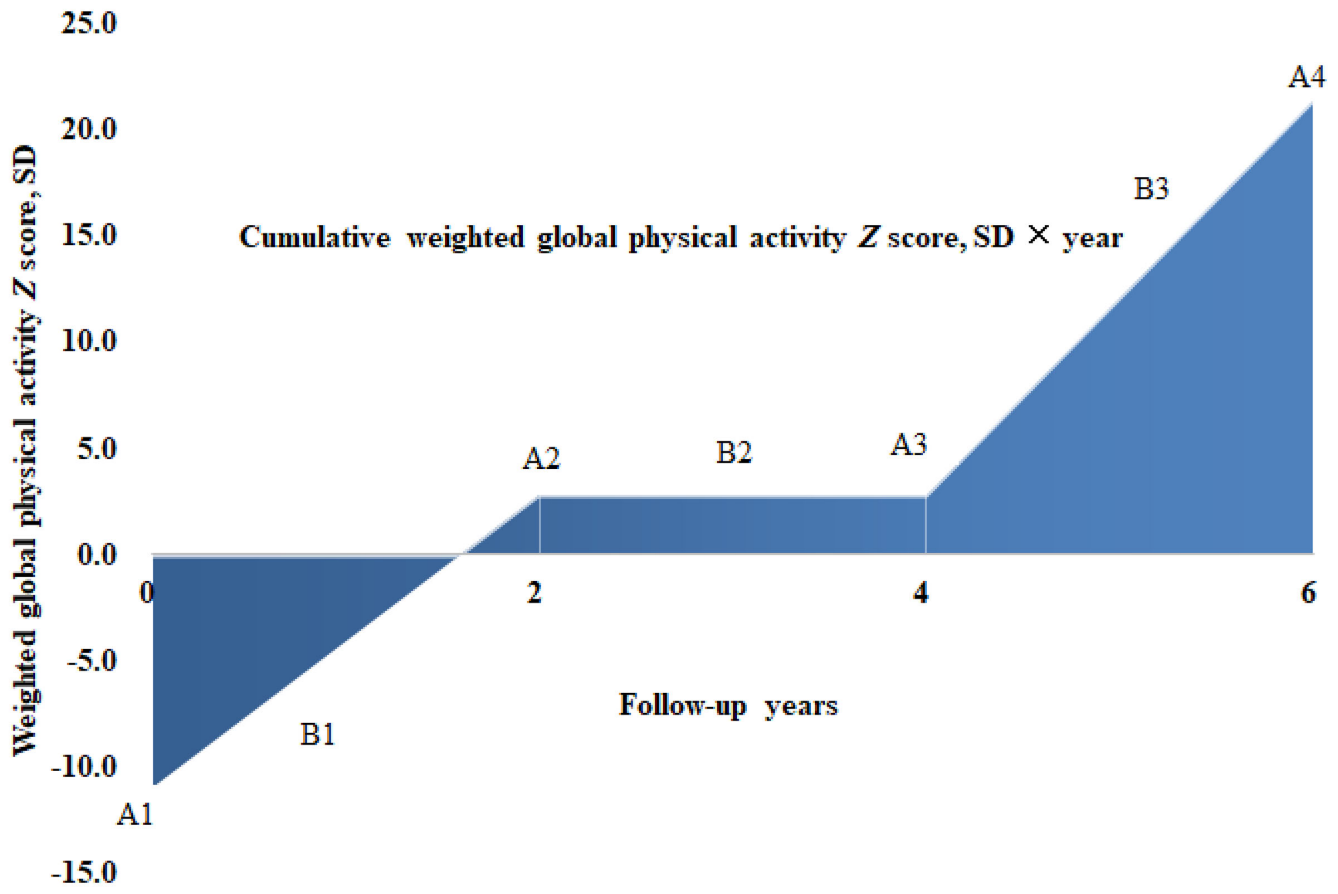
Cumulative physical activity ***Z*** score calculation for 1 participant across 4 visits over a 6-year span in the ELSA. A1, A2, A3, A4 denotes weighted global physical activity ***Z*** score of 4 visits at wave 1 (year 2002), wave 2 (year 2004), wave 3 (year 2006) and wave 4 (year 2008), while B1, B2 and B3 indicates average activity ***Z*** score between consecutive visits of A1, A2, A3 and A4, respectively. According to trapezoid rule, the cumulative weighted activity ***Z*** score (SD × year), denoted as area under the curve in color blue, could be calculated as (B1 × 2y + B2 × 2y + B3 × 2y), with same approach applied to calculate cumulative mild, moderate and vigorous physical activity ***Z*** score over a 6-year span at the level of participants.

We used weighted ***Z*** score of physical activity over a 6-year span from waves 1 to 4 to evaluate activity trajectories and cumulative participation. For physical activity trajectories, the group-based trajectory modeling (GBTM) was used to identify potential trajectories of participation in global, mild, moderate and vigorous activities, respectively. The GBTM used maximum likelihood estimation to identify participants sharing similar trajectory (22). It can handle data distributions including censored normal, Poisson and Bernoulli, and we used censored normal. Trajectory group of the highest probability was determined for each participant, which was then included in further multivariate analysis. We used SAS Proc Traj to fit GBTM models, with details described in **Supplemental Methods** (23). For cumulative participation, we used area under the curve (AUC) to calculate cumulative weighted physical activity ***Z*** score, based on Trapezoid rule (24). Details for AUC calculation were presented in **Figure 1**. Quintiles of cumulative weighted activity ***Z*** score were included in analysis as a numerical variable to perform linear trend test and mediation analysis.

Linear regression model was applied to assess associations of physical activity trajectories on subsequent BMI and waist circumference measured at wave 4. Cox regression was utilized to assess association of physical activity on risk of incident DM. Proportional hazard assumption was evaluated using weighted Schoenfeld residuals, with violated covariates included as model terms of interaction with time (25). To address ties in failure time, the method based on exact conditional probability under the proportional hazards assumption was applied (26). Hazard ratios (HR) and 95% Wald’s confidence intervals (CI) were reported. Observations with missing values were excluded from analysis.

Mediation analysis was performed to evaluate the role of obesity in association between cumulative weighted activity ***Z*** score quintiles and incident DM. We included two mediators to represent the obesity status, including BMI and waist circumference measured at wave 4. We also assessed the mediating role of change in BMI from waves 0 to 4. As a result, three mediation models were built. Each mediation model comprised of two separate models:

1) the mediator model, denoted as:

X+C→M

where X represented quintiles of cumulative weighted global physical activity ***Z*** score (numerical) and C represented vector of covariates including sex, age, ethnicity, education, cohabitation status, mobility status, current smoking, alcohol consumption, depressive symptoms, hypertension, prediabetes status, stroke, cardiovascular diseases, chronic lung diseases and cancer, as well as BMI in wave 0 (when the mediator was change in BMI). M represented hypothesized mediators. After assessing normality distribution for BMI, change in BMI, and waist circumference, linear regression was used for fitting the mediator model;

2) the response model, denoted as:

X+M+C→Y

where new variable Y represented recorded incident DM from waves 4 to 9, with meaning for X, M and C remaining the same. Considering the incident DM of binary type, binary logistic regression was used for fitting the response model, with exponential transformed coefficient equivalent to odds ratio (OR). Quasi-Bayesian approximation method was used to perform significance test for mediating effects, based on 2000 Monte Carlo replications (27).

Several sensitivity analyses were conducted. Firstly, we respectively identified trajectories of mild, moderate and vigorous activities, as well as calculated cumulative participation ***Z*** score. Then we evaluated associations between these sub-domain physical activity participation and outcomes. Secondly, we performed stratified analyses according to sex, age group (≥ 65 or <65 years), and overweight status (BMI < 25 or ≥ 25 kg/m^2^). Thirdly, we excluded participants suffered any difficulties in activities of daily living during waves 1 to 4, and participants developed incident DM within two years after wave 4, to address reverse causation (28). Finally, we analyzed association between physical activity and incident DM recorded in 2 to 6 years after wave 4, to explore the potential time range of protective tole by physical activity on incident DM.

All statistical analyses were conducted using SAS 9.4 (SAS Institute, Cary, NC), and the package mediation in R language 3.6.2 (R Foundation, Vienna, Austria), with two-tailed alpha of 0.05 referred as statistically significant level.

## Results

### Study Population

A total of 9757 diabetes-free participants from the ELSA were included for analysis. Detailed process of participant selection was shown in **Supplemental Figure 2**.

Among the included participants, 4243 (43.5%) participants were male, and the mean age was 58.9 ± 10.3 years. Differences were observed in most characteristics between activity trajectories, with details summarized in **Table 1**.

**Table 1:** Baseline characteristics of study population according to physical activity trajectories.

Characteristics^a^	AllN=9757	Initially low then improvingN=1868	Persistently lowN=3037	Initially high then decliningN=325	Persistently moderateN=2489	Persistently highN=2038	P value^b^
Male (%)	4243 (43.5%)	923 (49.4%)	1202 (39.6%)	139 (42.8%)	988 (39.7%)	991 (48.6%)	<0.001
Age (years)	58.9 ± 10.3	52.4 ± 7.8	59.1 ± 11.7	66.1 ± 10.7	61.9 ± 9.5	59.3 ± 8.2	<0.001
White (%)	9445 (96.8%)	1787 (95.7%)	2888 (95.1%)	316 (97.2%)	2442 (98.1%)	2012 (98.7%)	<0.001
High level of education (%)	2444 (25.0%)	48 (2.6%)	222 (7.3%)	90 (27.7%)	940 (37.8%)	1144 (56.1%)	<0.001
Living alone (%)	1879 (19.3%)	46 (2.5%)	492 (16.2%)	106 (32.6%)	759 (30.5%)	476 (23.4%)	<0.001
Current smoking (%)	980 (10.0%)	29 (1.6%)	245 (8.1%)	56 (17.2%)	426 (17.1%)	224 (11.0%)	<0.001
Drinking ≥ once per week (%)	3800 (38.9%)	92 (4.9%)	498 (16.4%)	188 (57.8%)	1545 (62.1%)	1477 (72.5%)	<0.001
Hypertension (%)	3087 (31.6%)	64 (3.4%)	688 (22.7%)	192 (59.1%)	1272 (51.1%)	871 (42.7%)	<0.001
Prediabetes (%)	1028 (10.5%)	19 (1.0%)	177 (5.8%)	62 (19.1%)	412 (16.6%)	358 (17.6%)	<0.001
Cardiovascular disease (%)	508 (5.2%)	16 (0.9%)	171 (5.6%)	38 (11.7%)	180 (7.2%)	103 (5.1%)	<0.001
Chronic lung disease (%)	301 (3.1%)	5 (0.3%)	120 (4.0%)	20 (6.2%)	111 (4.5%)	45 (2.2%)	<0.001
Cancer (%)	306 (3.1%)	6 (0.3%)	68 (2.2%)	22 (6.8%)	114 (4.6%)	96 (4.7%)	<0.001
Body mass index (kg/m^2^)	27.2 ± 4.4	27.2 ± 4.4	28.3 ± 5.1	27.6 ± 4.3	27.3 ± 4.4	26.5 ± 3.9	<0.001
Waist circumference (cm)	91.3 ± 12.7	92.6 ± 12.3	93.8 ± 13.5	92.3 ± 12.8	91.2 ± 12.6	89.9 ± 12.4	<0.001
Systolic blood pressure (mm Hg)	140.1 ± 19.4	137.3 ± 18.6	145.8 ± 21.3	142.8 ± 20.0	140.4 ± 19.3	136.6 ± 17.7	<0.001
Diastolic blood pressure (mm Hg)	77.5 ± 12.0	76.7 ± 12.2	77.9 ± 12.4	77.4 ± 11.4	77.8 ± 12.3	77.0 ± 11.6	0.193
Cumulative weighted global physical activity ***Z*** score (SD × year)	13.0 ± 56.7	-12.2 ± 28.2	-42.1 ± 17.8	16.6 ± 24.5	26.4 ± 19.8	101.2 ± 22.8	<0.001

a Data are presented as mean ± SD, n (%).

b P value reported for differences between trajectory groups using analysis of variance, or chi-square test.

### Long-Term Physical Activity Trajectories

**Figure 2:**
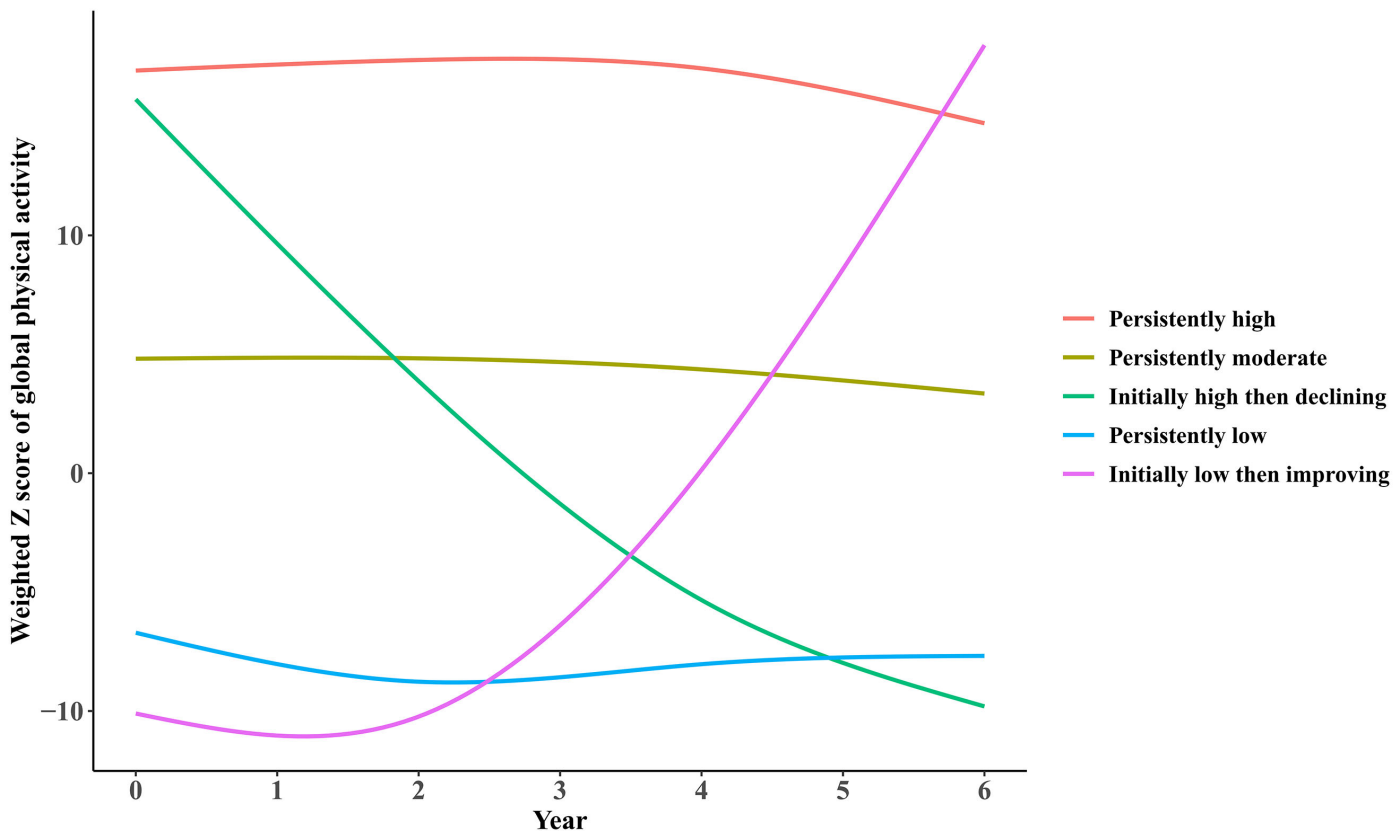
Trajectories of participation in global physical activities by participants from the ELSA over a 6-year span.

As shown in **Figure 2**, 5 trajectories of global physical activity were identified, including: 1) persistently low trajectory (N=3037), with constantly low participation; 2) initially low then improving (N=1868), with initially low participation but turned to elevating afterwards; 3) initially high then declining (N=325), with initially high participation but turned to declining afterwards; 4) persistently moderate (N=2489), with constantly intermediate participation; 5) persistently high (N=2038), with highly active participation throughout the span.

### Association of Physical Activity Participation on Obesity

As illustrated in **Table 2**, significant associations were observed of global physical activity trajectories and cumulative participation on subsequent obesity. Compared with the persistently low trajectory, participants in trajectories of initially low then improving, persistently moderate and high had significantly lower BMI of 1.121 kg/m^2^ (95% CI: 0.777 to 1.465, *P <*0.001), 0.688 kg/m^2^ (95% CI: 0.345 to 1.031, *P* < 0.001), and 1.441 kg/m^2^ (95% CI: 1.070 to 1.813, *P <*0.001), respectively. And those with initially high then declining trajectory showed no significant differences compared with persistently low trajectory. Similar results were observed for waist circumference. Compared with the persistently low trajectory, participants in trajectories of initially low then improving, persistently moderate and high had significantly lower waist circumference of 3.183 cm (95% CI: 2.351 to 4.015, *P <*0.001), 2.367 cm (95% CI: 1.544 to 3.191, *P* < 0.001), and 4.740 cm (95% CI: 3.846 to 5.634, *P <*0.001), respectively.

**Table 2:** Association of long-term physical activity participation on subsequent obesity assessed by BMI and waist circumference.

Physical activity participation	BMI (kg/m^2^)		Waist circumference (cm)	
	β (95% Cl)^a^	P value	β (95% Cl)	P value
**Global physical activity trajectories**				
Persistently low	Reference		Reference	
Initially low then improving	-1.121 (-1.465, -0.777)	<0.001	-3.183 (-4.015, -2.351)	<0.001
Initially high then declining	-0.134 (-0.817, 0.549)	0.701	-1.487 (-3.101, 0.127)	0.071
Persistently moderate	-0.688 (-1.031, -0.345)	<0.001	-2.367 (-3.191, -1.544)	<0.001
Persistently high	-1.441 (-1.813, -1.070)	<0.001	-4.740 (-5.634, -3.846)	<0.001
**Cumulative weighted global physical activity participation *Z* score (SD × year)**				
Quintile 1	Reference		Reference	
Quintile 2	-0.810 (-1.179, -0.441)	<0.001	-2.620 (-3.506, -1.735)	<0.001
Quintile 3	-0.994 (-1.387, -0.601)	<0.001	-2.873 (-3.818, -1.927)	<0.001
Quintile 4	-1.396 (-1.786, -1.006)	<0.001	-4.221 (-5.161, -3.281)	<0.001
Quintile 5	-1.860 (-2.276, -1.444)	<0.001	-5.746 (-6.748, -4.745)	<0.001
Test for linear trend	-0.436 (-0.533, -0.339)	<0.001	-1.338 (-1.572, -1.104)	<0.001
Per 10 units increment	-0.109 (-0.133, -0.085)	<0.001	-0.348 (-0.406, -0.291)	<0.001

a Adjusted covariates included sex, age, ethnicity, education, cohabitation status, mobility status, current smoking, alcohol consumption, depressive symptoms, overweight status, hypertension, prediabetes status, stroke, cardiovascular diseases, chronic lung diseases and cancer.

As for cumulative physical activity participation, it was found that elevated participation was associated with both lower BMI and waist circumference, with each 10 units increment in cumulative weighted global activity ***Z*** score associated with decreased BMI of 0.109 kg/m^2^ (95% CI: 0.085 to 0.133, *P <*0.001) and waist circumference of 0.348 cm (95% CI: 0.291 to 0.406, *P <*0.001).

### Association Between Physical Activity Participation and Incident DM

During a 10-year span, 670 incident DM cases were reported, with results summarized in **Table 3**. Compared with the persistently low trajectory, participants in initially low then improving, persistently moderate and high trajectories had significant lower DM risk, with HR of 0.41 (95% CI: 0.32 to 0.53, *P <*0.001), 0.70 (95% CI: 0.56 to 0.89, *P* =0.004) and 0.49 (95% CI: 0.37 to 0.65, *P <*0.001), respectively. By contrast, DM risk in initially high then declining trajectory showed no significant differences with persistently low trajectory. For cumulative activity participation, elevated cumulative weighted global activity ***Z*** score was also associated with lower DM risk, with each quintile and 10 units increment corresponding to HR of 0.76 (95% CI: 0.71 to 0.82, *P <*0.001) and 0.94 (95% CI: 0.92 to 0.95, *P <*0.001), respectively.

**Table 3:** Association of long-term physical activity participation on subsequent incident DM.

Physical activity participation	Events/Total	Model 1^a^		Model 2^b^	
		HR (95% CI)	P value	HR (95% CI)	P value
**Global physical activity trajectories**					
Persistently low	282/3037	Reference		Reference	
Initially low then improving	90/1868	0.34 (0.26, 0.43)	<0.001	0.41 (0.32, 0.53)	<0.001
Initially high then declining	20/325	1.16 (0.73, 1.84)	0.531	0.75 (0.47, 1.20)	0.226
Persistently moderate	170/2489	1.04 (0.84, 1.29)	0.736	0.70 (0.56, 0.89)	0.004
Persistently high	108/2038	0.67 (0.52, 0.87)	0.003	0.49 (0.37, 0.65)	<0.001
**Cumulative weighted global physical activity participation Z score (SD × year)**					
Quintile 1	182/2015	Reference		Reference	
Quintile 2	136/1888	0.68 (0.54, 0.87)	0.002	0.57 (0.45, 0.73)	<0.001
Quintile 3	140/1951	0.89 (0.70, 1.12)	0.321	0.51 (0.40, 0.66)	<0.001
Quintile 4	107/1949	0.58 (0.45, 0.75)	<0.001	0.38 (0.29, 0.50)	<0.001
Quintile 5	105/1954	0.58 (0.44, 0.78)	<0.001	0.34 (0.25, 0.46)	<0.001
Test for linear trend	–	0.87 (0.82, 0.93)	<0.001	0.76 (0.71, 0.82)	<0.001
Per 10 units increment	–	0.97 (0.95, 0.98)	<0.001	0.94 (0.92, 0.95)	<0.001

a Adjusted for age × time, sex, ethnicity and education.

b Additionally adjusted for cohabitation status, mobility status, current smoking, alcohol consumption, depressive symptoms, overweight status, hypertension, prediabetes status, stroke, cardiovascular diseases, chronic lung diseases and cancer.

### Mediation Analysis for Obesity

**Figure 3:**
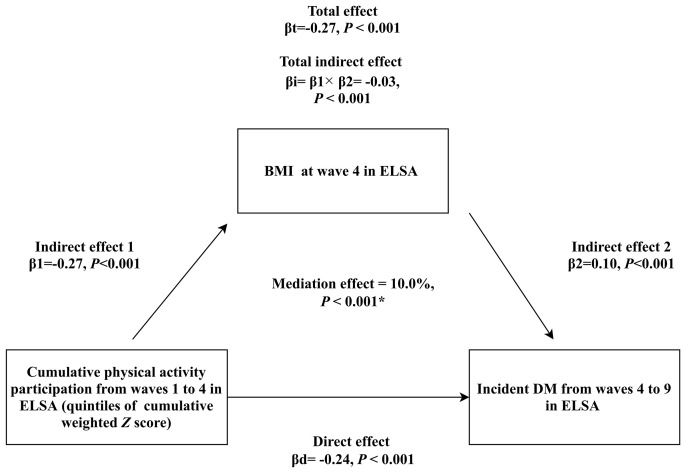
Mediation analysis assessing the mediating role of BMI measured at wave 4 in the association of cumulative weighted global physical activity ***Z*** score quintiles on subsequent incident DM. βd: coefficient for direct association of cumulative weighted global physical activity ***Z*** score quintiles on subsequent incident DM. βi: coefficient for total indirect association of BMI. βt: coefficient for total association of cumulative weighted global physical activity ***Z*** score quintiles on subsequent incident DM. ^*^: adjusted covariates included sex, age, ethnicity, education, cohabitation status, mobility status, current smoking, alcohol consumption, depressive symptoms, hypertension, prediabetes status, stroke, cardiovascular diseases, chronic lung diseases and cancer.

As shown in **Figures 3** **–** **5**, significant mediation roles by BMI, waist circumference, and change in BMI were identified in association between cumulative weighted global physical activity ***Z*** score quintiles and subsequent incident DM, after adjusting for other covariates. The total indirect effect through BMI (βi=-0.03, *P <*0.001), defined as product of indirect effect 1 (β1) and indirect effect 2 (β2) reached significance level, which mediated 10% (*P <*0.001) of overall association. Similarly, the total indirect effect through waist circumference (βi=-0.05, *P <*0.001) and change in BMI (βi=-0.02, *P <*0.001) were significant, mediating 17% (*P <*0.001) and 9% (*P <*0.001) of overall association, respectively.

**Figure 4:**
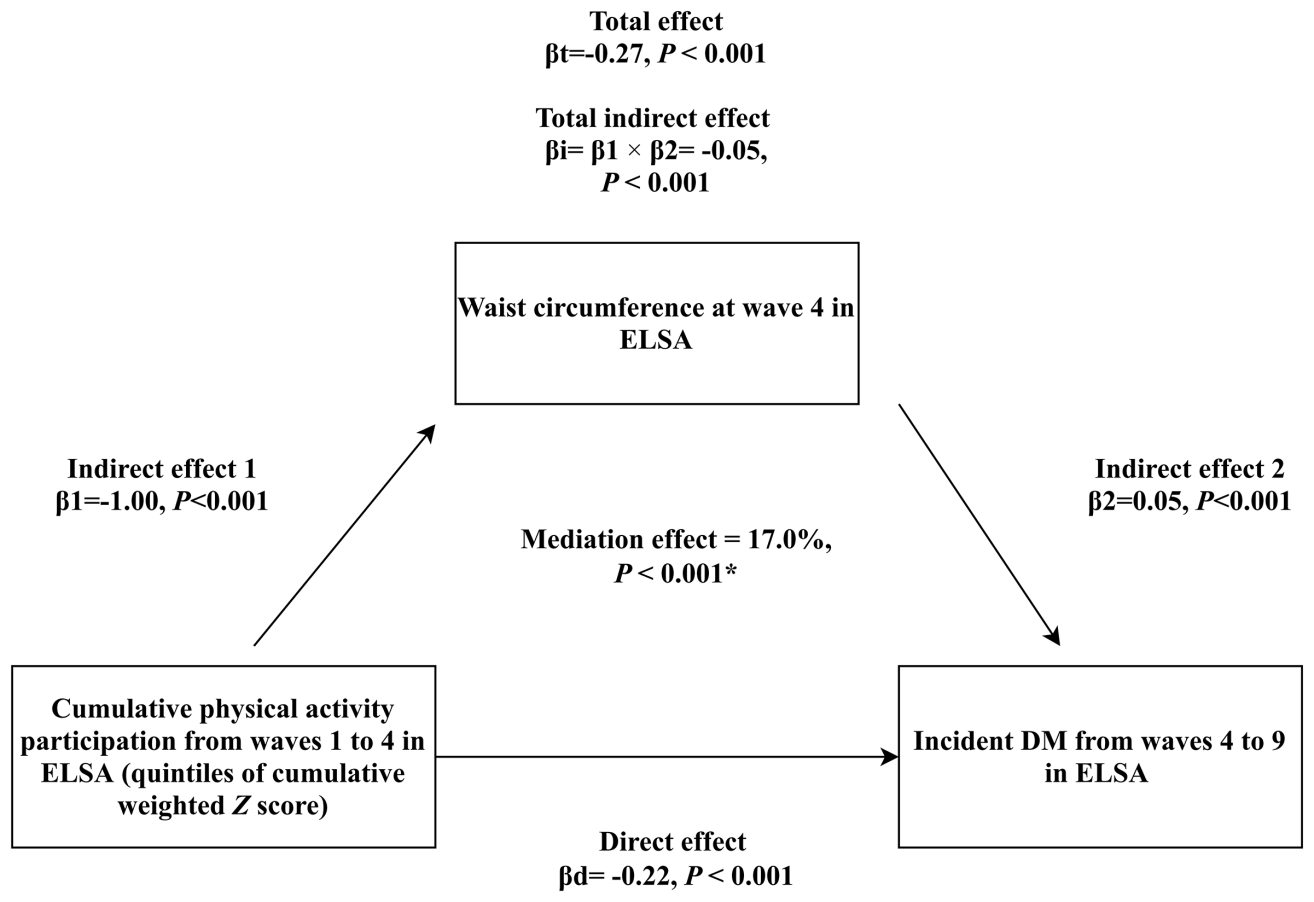
Mediation analysis assessing the mediating role of waist circumference measured at wave 4 in the association of cumulative weighted global physical activity ***Z*** score quintiles on subsequent incident DM. βd: coefficient for direct association of cumulative weighted global physical activity ***Z*** score quintiles on subsequent incident DM. βi: coefficient for total indirect association of waist circumference. βt: coefficient for total association of cumulative weighted global physical activity ***Z*** score quintiles on subsequent incident DM. ^*^: adjusted covariates included sex, age, ethnicity, education, cohabitation status, mobility status, current smoking, alcohol consumption, depressive symptoms, hypertension, prediabetes status, stroke, cardiovascular diseases, chronic lung diseases and cancer.

**Figure 5:**
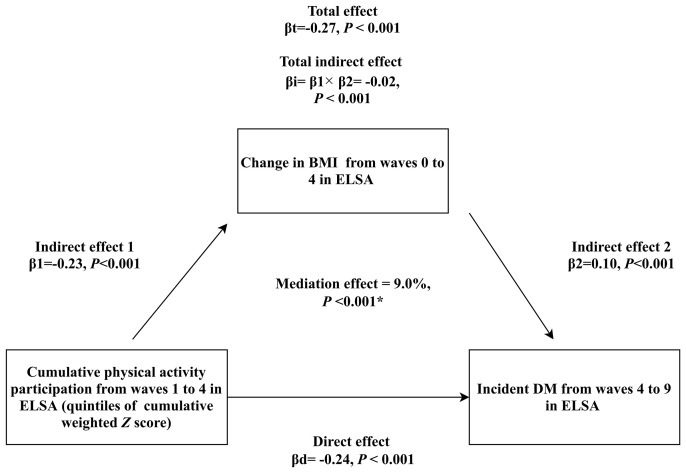
Mediation analysis assessing the mediating role of change in BMI from waves 0 to 4 in the association of cumulative weighted global physical activity ***Z*** score quintiles on subsequent incident DM. βd: coefficient for direct association of cumulative weighted global physical activity ***Z*** score quintiles on subsequent incident DM. βi: coefficient for total indirect association of change in BMI. βt: coefficient for total association of cumulative weighted global physical activity ***Z*** score quintiles on subsequent incident DM. ^*^: adjusted covariates included sex, age, ethnicity, education, cohabitation status, mobility status, current smoking, alcohol consumption, BMI in wave 0, depressive symptoms, hypertension, prediabetes status, stroke, cardiovascular diseases, chronic lung diseases and cancer.

### Sensitivity Analyses

Similar with global physical activity, 5 trajectories were identified for both mild and moderate activities, as shown in **Supplemental Figures 3, 4**, respectively. For vigorous activities, a total of 4 trajectories were identified, shown in **Supplemental Figure 5**.

Associations of sub-domain physical activity participation on obesity, and incident DM risk were presented in **Supplemental Tables 2, 3**, respectively. For obesity, consistent results with global activity participation were observed for all three mild, moderate and vigorous intensity activities. For incident DM, consistent results were mainly observed for moderate and vigorous intensity activities participation.

Stratified analyses by sex (**Supplemental Tables 4, 5**), age (**Supplemental Tables 6, 7**), and overweight status (**Supplemental Tables 8, 9**) showed that similar results were consistently observed. After further excluding participants with difficulties in daily living activities or developed incident DM within two years after wave 4, our main results preserved, shown in **Supplemental Tables 10, 11**. For physical activity trajectories, significant associations with incident DM risk reduction in 2 to 6 years were consistently observed for the persistently moderate group, shown in **Supplemental Table 12**. The initially low then improving group was not associated with incident DM risk in 2 years but associated with lower incident DM risk in 4 to 6 years after wave 4, while no significant associations were observed for the initially high then declining and persistently high groups in this span.

## Discussion

Our study presented several major findings. Firstly, persistently active participation in physical activity was associated with a lower risk of subsequent incident DM. Secondly, for those with initially low activity, gradually improved participation was also associated with subsequent lower DM risk. These findings were encouraging, indicating that taking measures towards being physically active could never be too late for DM prevention. According to WHO recommendations, even for adults aged ≥ 65, health benefits could still be gained by maintaining adequate activity (29). And our findings further illustrated DM prevention benefits. Finally, both overall obesity assessed by BMI and central obesity by waist circumference significantly mediated association between activity and incident DM, with central obesity in particular. The implication was that obesity could be crucial to understand the link between activity and DM. According to previous studies, central obesity was as important as overall obesity in predicting DM risk, and our study further consolidated its significance by illustrating potential mediation role linking long-term activity with subsequent DM risk (30–32).

To our current knowledge, this is the first prospective study to investigate both trajectories and cumulative participation of physical activity over a 6-year span, and assess its association with subsequent 10-year incident DM, as well as the mediation role by obesity, with findings implicative of potential DM prevention significance from active physical participation.

Several previous studies also discussed association between long-term activity, obesity and DM. In a cohort study evaluating longitudinal trajectories of metabolic control from childhood to young adulthood in DM patients, researchers used GBTM approach to identify longitudinal trajectories of HbA1c and evaluated related factors (33). They found that elevated frequency of participating in physical activity was associated with lower probability of belonging to HbA1c increase trajectories, illustrating potential significance of being physically active (33). Another cohort study recruiting Chinese adults aged 20 to 80 years with impaired fasting glucose found that an inverse dose–response relationship between leisure-time physical activity and incident DM risk (34). The researchers also found that 19.2% incident DM could be avoided if those inactive participants could have engaged in WHO recommendation levels of leisure-time physical activity (34). In another study investigating association between BMI trajectories and DM among women aged between 30 and 100 years, researchers found that leisure time physical activity attenuated the general deleterious effect of obesity on DM, especially for women of the consistently non-obese trajectory (35). Although these findings showed similarity with ours, few of them directly evaluated long-term physical activity participation and the longitudinal association of on subsequent incident DM. In another 5-year cohort study of older adults aged ≥ 50 years in China, researchers assessed association between onset of DM and long-term physical activity trajectories (36). They found that, participants with longer cumulative years in participating physical activity experienced a later onset of obesity and DM, compared to their sedentary counterparts. They also evaluated the association of activity on risk of onset of obesity and DM, but failed to observe significant results (36). Despite that, the study indicated the significance of long-term active participation in physical activity in delaying onset of DM and obesity, which was consistent with our findings. Another study including South Koreans over 18 years old also found that both sufficient baseline physical activity level and its temporal increase were associated with a lower risk of incident DM, compared with those with temporal decrease in activity (37). Regardless of these shared findings in protective role of long-term active activity, our findings concerning the mediation role by BMI and waist circumference still require verification by further prospective studies. Although studies reported the mediation role by central obesity in linking genetic predisposition with incident DM risk, whether same mediation role maintained for long-term physical activity remain unresolved (38).

Our study found consistent association of moderate and vigorous intensity activities on incident DM, demonstrating significance of regular moderate-to-vigorous activities participation for DM prevention. The findings were also consistent with recommendations by ADA concerning DM prevention (39).

Our study possessed several strengths. Firstly, we simultaneously assessed trajectories and cumulation of participation in physical activity over a 6-year span, and prospectively assessed its associations with subsequent 10-year incident DM risk. Such design enabled stronger capability of drawing casual conclusions. Secondly, we used the GBTM approach to explore all possible pattern of physical activity trajectories over a 6-year span, instead of merely assuming monotonic trajectories. The approach could efficiently incorporate repeated measurements of activity participation at multiple timepoints, thus could address limitations by only considering activity participation at single time-point. Thirdly, our study evaluated the mediation role by obesity, which enabled better understanding of the observed association between long-term physical activity and DM. Finally, our study population was nationally-representative of community-dwelling adults aged ≥ 50 years in England, with large sample-size and long follow-up period.

Several limitations also required attention. Firstly, we only considered frequency when evaluating physical activity participation, without accounting for duration. Besides, only reported frequency was used, thus the issue of reporting bias remains non-neglectable. Secondly, the majority of participants included was of White ethnicity, which restricted generalization to other ethnicities. Thirdly, the challenging issue of reverse causation remains. Chances were that some previous diagnosed diseases or pre-clinical changes in metabolic status could impact regular physical activity participation, and lead us to biased conclusions. Nevertheless, considering the long follow-up period of our study, influence by reverse causation on our findings could be limited (40). Fourthly, due to data restrictions, we could not account for the potential impact by dietary pattern on incident DM. Giving the significant role by diet in incident DM prevention, such missing consideration could leave our findings to potential bias (18). Finally, due to the nature of observational study, influence by unmeasured confounding factors on our results could not be eliminated, impeding further steps towards conclusive casual relationships (41).

In summary, we found that for middle aged and older adults, both gradually improved and persistently active participation in physical activity were associated with subsequent lower risk of incident DM, with obesity playing a potential mediator. Strategies focusing on improving and maintaining active participation in physical activity might be beneficial from DM prevention perspectives.

## Data Availability Statement

Original survey dataset from the ELSA are freely available to all bonafide researchers. Access to data can be obtained by visiting their websites (https://www.elsa-project.ac.uk/). The data can also be obtained on request (xiewuxiang@hsc.pku.edu.cn).

## Ethics Statement

The ELSA was approved by the London Multicentre Research Ethics Committee (MREC/01/2/91). The patients/participants provided their written informed consent to participate in this study.

## Author Contributions

CL contributed to formal analysis and writing original draft. YM and RH contributed to data curation and manuscript editing efforts. FZ and WX conceptualized the study design and funding acquisition, as well as manuscript reviewing and editing efforts. All authors had full access to the data in the study and can take responsibility for the integrity of the data and the accuracy of the data analysis. FZ and WX and are the guarantors. The corresponding author attests that all listed authors meet authorship criteria and that no others meeting the criteria have been omitted. All authors contributed to the article and approved the submitted version.

## Funding

The present study was supported by the National Natural Science Foundation of China (project no. 81974490) and 2019 Irma and Paul Milstein Program for Senior Health Research Project Award. The funders had no role in the study design; the collection, analysis, and interpretation of data; the writing of the manuscript; or the decision to submit the article for publication.

## Conflict of Interest

The authors declare that the research was conducted in the absence of any commercial or financial relationships that could be construed as a potential conflict of interest.

## Publisher’s Note

All claims expressed in this article are solely those of the authors and do not necessarily represent those of their affiliated organizations, or those of the publisher, the editors and the reviewers. Any product that may be evaluated in this article, or claim that may be made by its manufacturer, is not guaranteed or endorsed by the publisher.
