# Supplementary material for: Long-Term Physical Activity Participation and Subsequent Incident Type 2 Diabetes Mellitus: A Population-Based Cohort Study
Source: Front Endocrinol (Lausanne). 2021 Nov 30;12:769549. doi: 10.3389/fendo.2021.769549 (PMC8669796; doi:10.3389/fendo.2021.769549)
Supplement: Supplementary file 1 [file DataSheet_1_v1.pdf]

Long-term physical activity participation and subsequent incident type 2 diabetes mellitus: a population-based cohort study

Chenglong Li, Bachelor [1, 2, 3]; Yanjun Ma, Bachelor [1, 2, 3]; Rong Hua, Bachelor [1, 2, 3]; Fanfan Zheng, PhD [4*]; Wuxiang Xie, PhD [1, 2, 3*]

Affiliations:

[1] Peking University Clinical Research Institute, Peking University First Hospital, Beijing, China;

[2] PUCRI Heart and Vascular Health Research Center at Peking University Shougang Hospital, Beijing, China;

[3] Key Laboratory of Molecular Cardiovascular Sciences (Peking University), Ministry of Education, Beijing, China;

[4] School of Nursing, Peking Union Medical College, Beijing, China.

Correspondence:

Fanfan Zheng, School of Nursing, Peking Union Medical College, 33 Ba Da Chu Road, Shijingshan District, 100144, Beijing, China; Email: fanfan@bjmu.edu.cn;

Wuxiang Xie, Peking University Clinical Research Institute, Peking University First Hospital, No. 8 Xishiku Street, Xicheng District, 100034, Beijing, China; E-mail: xiewuxiang@hsc.pku.edu.cn.

Supplemental methods

Supplemental tables

Supplemental Table 1. Physical activity intensity categories, activity types, MET values, codes, and MET weights.

Supplemental Table 2. Association of long-term sub-domain physical activity participation on subsequent obesity assessed by BMI and waist circumference.

Supplemental Table 3. Association of long-term sub-domain physical activity participation on subsequent incident DM.

Supplemental Table 4. Association of long-term physical activity participation on subsequent obesity assessed by BMI and waist circumference, stratified by sex.

Supplemental Table 5. Association of long-term physical activity participation on subsequent incident DM, stratified by sex.

Supplemental Table 6. Association of long-term physical activity participation on subsequent obesity assessed by BMI and waist circumference, stratified by age.

Supplemental Table 7. Association of long-term physical activity participation on subsequent incident DM, stratified by age.

Supplemental Table 8. Association of long-term physical activity participation on subsequent obesity assessed by BMI and waist circumference, stratified by BMI.

Supplemental Table 9. Association of long-term physical activity participation on subsequent incident DM, stratified by BMI.

Supplemental Table 10. Association of long-term physical activity participation on

subsequent obesity assessed by BMI and waist circumference, after excluding participants reported any difficulties in activities of daily living during waves 1 to 4, or developed incident DM within two years after wave 4.

Supplemental Table 11. Association of long-term physical activity participation on subsequent incident DM, after excluding participants reported any difficulties in activities of daily living during waves 1 to 4, or developed incident DM within two years after wave 4.

Supplemental Table 12. Association of long-term physical activity participation on subsequent incident DM recorded in 2 to 6 years after wave 4.

Supplemental figures

Supplemental Figure 1. Study timeline and design.

Supplemental Figure 2. Flow chart of participants selection.

Supplemental Figure 3. Trajectories of participation in mild intensity physical activities by participants from the ELSA over a 6-year span.

Supplemental Figure 4.Trajectories of participation in moderate intensity physical activities by participants from the ELSA over a 6-year span.

Supplemental Figure 5. Trajectories of participation in vigorous intensity physical activities by participants from the ELSA over a 6-year span.

Supplemental Methods

BMI and waist circumference measurement

All participants were eligible to have their height and weight measured. If the nurse thought the measurement was likely to be more than 2 cm (3/4 inch) from the true figure for height or more than 1 kg (2 lbs.) from the true figure for weight, it was considered unreliable and they were asked to code it as such. Using confirmed reliable measured standing height and weight, BMI was calculated by dividing weight in kilograms by height in meters squared. For waist circumference, typically two measurements were conducted for each visit, unless the second measurement differed from the first by 3 cm or more, then the nurse was prompted to either amend one of the previous responses if a mistake had been made entering a measurement, or to take a third measurement. If the nurse believed that the measurements they took were 0.5 cm more or less than the true measurement because of problems encountered (e.g. clothing the respondent was wearing), this was considered unreliable. Only reliable measurements were used to calculate mean waist circumference.

Physical activity assessment

The ELSA used three questions to measure participation in physical activities of mild, moderate and vigorous intensity. Unified expression of these questions was "We would like to know the type and amount of physical activity involved in your daily life. Do you take part in sports or activities that are vigorous/moderately energetic/mildly energetic more than once a week, or once a week, or one to three times a month, or hardly ever or never ?". A card was presented for participants when

being asked about these questions, with examples of different types of activities provided. Detailed examples included: 1) laundry, home repairs for mild intensity; 2) gardening, cleaning the car, walking at a moderate pace, dancing, floor or stretching exercises for moderate intensity; 3) running or jogging, swimming, cycling, aerobics or gym workout, tennis, digging with a spade or shovel for vigorous intensity. Participants were also allowed to list additional examples and required to decide which of the three categories (vigorous, moderate and mild) could best match the activity.

GBTM modeling

The GBTM can fit non-monotonic trajectories and support multiple trajectory shapes including linear, quadratic and cubic. It also allows specification of number of trajectory groups before fitting the model. We selected number of groups from 3 to 7 and compared model fit statistics of the Bayesian information criterion (BIC) of different trajectory models to determine the most optimal number of trajectory groups. Then, we determined that modeling 5 trajectory groups was appropriate for global, mild and moderate physical activity trajectories modeling, while 4 trajectory groups for vigorous physical activity trajectories modeling.

We further evaluated different trajectory shapes for each trajectory group by testing the null hypothesis that the shape parameter for the group equals zero. We also used graphics of trajectory group means to help determine which shape best fit each trajectory group. After the procedure, we determined that the best 5-group trajectory model for global, mild and moderate physical activity consisted of 2 cubic and 3

linear trajectories, while the best 4-group model for vigorous physical activity consisted of 3 cubic and 1 linear trajectories. Then the estimated trajectory groups membership was included as the independent variable for further multivariate analysis.

COX regression modeling

Association between physical activity trajectories, cumulative physical activity participation ***Z*** score and incident DM risk was evaluated using proportional hazard regression (Cox regression) model. We evaluated proportional hazard assumption for all included covariates using weighted Schoenfeld residuals, and addressed violation of assumption by including covariates as interaction term with time scale variable (years from wave 4 to first occurrence of event in interest or censoring). After assessment, the covariate age was identified of significant correlation with time scale variable, and we included the interaction term of age × time for all Cox regression models adjusted for age.

Supplemental Table 1. Physical activity intensity categories, activity types, MET values, codes, and MET weights.

Intensity	Activity type [a]	MET [b]	Code [c]	MET weight [d]
Low	Laundry	2.15	Mean of 05090, and 05095	2.3
Low	Home repairs	2.50	06126	
Moderate	Gardening	3.80	08245	
Moderate	Cleaning the car	3.50	05020	
Moderate	Walking at a moderate pace	5.30	17082	
Moderate	Dancing	5.85	Mean of 03010, 03014, 03019, 03020, 03025, 03030, 03031, 03038, 03040,03050, 03060	4.4
Moderate	Floor or stretching exercises	3.50	05130	

Vigorous	Running or jogging	6.70	Mean of 12010, 12020, 12025, 12027, 12150	
Vigorous	Swimming	7.20	Mean of 18230, 18240, 18310	
Vigorous	Cycling	6.80	01011	
Vigorous	Aerobics or gym workout	7.30	03015	7.2
Vigorous	Tennis	7.10	Mean of 15675, 15680, 15690	
Vigorous	Digging with a spade or shovel	7.80	08052	

[a] Activity type was selected based on examples showed to participants when investigating frequency of physical activity.

[b] MET: metabolic equivalent of tasks. MET estimates were derived according to 2011 Compendium of Physical Activities.

[c] Code represented exact type of activities, for activity corresponding to multiple potential types, we used mean of MET values from these activities.

[d] Mean of all activity types' MET was used to calculate MET weights for low, moderate, and vigorous intensity activities, respectively.

Supplemental Table 2. Association of long-term sub-domain physical activity participation on subsequent obesity assessed by BMI and waist circumference.

Sub-domain physical activity participation	BMI (kg/m^2)		Waist circumference (cm)	
	β (95% CI)[a]	P value	β (95% CI)	P value
**Mild intensity physical activity trajectories**				
Persistently low	Reference		Reference	
Initially low then improving	-0.635 (-1.194, -0.076)	0.026	-1.995 (-3.327, -0.663)	0.003
Initially high then declining	-0.775 (-1.832, 0.282)	0.151	-2.228 (-4.681, 0.225)	0.075
Persistently high	-1.169 (-1.760, -0.577)	<0.001	-4.059 (-5.467, -2.652)	<0.001
Initially improving then declining	-1.173 (-2.022, -0.324)	0.007	-3.822 (-5.832, -1.813)	<0.001
**Cumulative mild intensity physical activity participation *Z* score (SD × year)**				
Per 1 unit increment	-0.090 (-0.125, -0.055)	<0.001	-0.303 (-0.388, -0.219)	<0.001
**Moderate intensity physical activity trajectories**				
Persistently low	Reference		Reference	
Initially low then improving	-1.653 (-2.080, -1.225)	<0.001	-3.945 (-4.970, -2.921)	<0.001
Initially high then declining	-1.125 (-1.856, -0.394)	0.003	-2.899 (-4.622, -1.177)	<0.001
Persistently high	-2.154 (-2.605, -1.702)	<0.001	-5.478 (-6.551, -4.405)	<0.001
Initially improving then declining	-1.301 (-1.971, -0.632)	<0.001	-3.304 (-4.895, -1.714)	<0.001

**Cumulative moderate intensity physical activity participation *Z* score (SD × year)**				
Per 1 unit increment	-0.146 (-0.179, -0.113)	<0.001	-0.396 (-0.476, -0.317)	<0.001
**Vigorous intensity physical activity trajectories**				
Persistently low	Reference		Reference	
Initially low then improving	-0.878 (-1.174, -0.581)	<0.001	-2.677 (-3.394, -1.960)	<0.001
Initially high then declining	-0.129 (-0.520, 0.261)	0.516	-0.900 (-1.841, 0.041)	0.061
Persistently high	-0.968 (-1.289, -0.647)	<0.001	-3.592 (-4.370, -2.814)	<0.001
**Cumulative vigorous intensity physical activity participation *Z* score (SD × year)**				
Per 1 unit increment	-0.091 (-0.115, -0.067)	<0.001	-0.309 (-0.367, -0.251)	<0.001

[a] Adjusted covariates included sex, age, ethnicity, education, cohabitation status, mobility status, current smoking, alcohol consumption, depressive symptoms, overweight status, hypertension, prediabetes status, stroke, cardiovascular diseases, chronic lung diseases and cancer.

Supplemental Table 3. Association of long-term sub-domain physical activity participation on subsequent incident DM.

Physical activity participation	Events/Total	Model 1[a]	
		HR (95% CI)	P value
**Mild intensity physical activity trajectories**			
Persistently low	53/577	Reference	
Initially low then improving	241/3539	0.65 (0.47, 0.90)	0.010
Initially high then declining	12/228	0.80 (0.41, 1.55)	0.506
Persistently high	336/5092	0.74 (0.52, 1.06)	0.103
Initially improving then declining	28/321	1.01 (0.61, 1.67)	0.981
**Cumulative mild intensity physical activity participation *Z* score (SD × year)**			
Per 1 unit increment	-	0.96 (0.94, 0.99)	0.004
**Moderate intensity physical activity trajectories**			
Persistently low	119/1147	Reference	
Initially low then improving	217/3333	0.47 (0.36, 0.60)	<0.001
Initially high then declining	29/385	0.94 (0.61, 1.45)	0.780
Persistently high	278/4478	0.51 (0.39, 0.68)	<0.001
Initially improving then declining	27/414	0.63 (0.40, 0.98)	0.039
**Cumulative moderate intensity physical activity participation *Z* score (SD × year)**			

Per 1 unit increment	-	0.94 (0.91, 0.96)	<0.001
**Vigorous intensity physical activity trajectories**			
Persistently low	442/5311	Reference	
Initially low then improving	92/1973	0.45 (0.35, 0.57)	<0.001
Initially high then declining	52/894	0.69 (0.51, 0.93)	0.015
Persistently high	84/1579	0.59 (0.45, 0.76)	<0.001
**Cumulative vigorous intensity physical activity participation *Z* score (SD × year)**			
Per 1 unit increment	-	0.94 (0.92, 0.96)	<0.001

[a] Adjusted for age × time, sex, ethnicity and education, cohabitation status, mobility status, current smoking, alcohol consumption, depressive symptoms, overweight status, hypertension, prediabetes status, stroke, cardiovascular diseases, chronic lung diseases and cancer.

Supplemental Table 4. Association of long-term physical activity participation on subsequent obesity assessed by BMI and waist circumference, stratified by sex.

Physical activity participation	BMI (kg/m^2)		Waist circumference (cm)	
	β (95% CI)[a]	P value	β (95% CI)	P value
**Men**				
**Global physical activity trajectories**				
Persistently low	Reference		Reference	
Initially low then improving	-0.814 (-1.254, -0.374)	<0.001	-3.306 (-4.448, -2.164)	<0.001
Initially high then declining	-0.115 (-1.085, 0.854)	0.816	-2.790 (-5.218, -0.363)	0.024
Persistently moderate	-0.747 (-1.244, -0.250)	0.003	-2.168 (-3.447, -0.889)	<0.001
Persistently high	-1.429 (-1.939, -0.919)	<0.001	-4.589 (-5.906, -3.272)	<0.001
**Cumulative weighted global physical activity participation *Z* score (SD × year)**				
Per 10 units increment	-0.099 (-0.130, -0.067)	<0.001	-0.319 (-0.401, -0.238)	<0.001
**Women**				
**Global physical activity trajectories**				
Persistently low	Reference		Reference	
Initially low then improving	-1.421 (-1.932, -0.910)	<0.001	-3.159 (-4.350, -1.967)	<0.001
Initially high then declining	-0.134 (-1.076, 0.807)	0.780	-0.528 (-2.693, 1.636)	0.632
Persistently moderate	-0.645 (-1.113, -0.176)	0.007	-2.420 (-3.504, -1.336)	<0.001

Persistently high	-1.444 (-1.971, -0.916)	<0.001	-4.771 (-5.996, -3.546)	<0.001
**Cumulative weighted global physical activity participation *Z* score (SD × year)**				
Per 10 units increment	-0.117 (-0.151, -0.082)	<0.001	-0.369 (-0.449, -0.288)	<0.001

[a] Adjusted covariates included sex, age, ethnicity, education, cohabitation status, mobility status, current smoking, alcohol consumption, depressive symptoms, overweight status, hypertension, prediabetes status, stroke, cardiovascular diseases, chronic lung diseases and cancer.

Supplemental Table 5. Association of long-term physical activity participation on subsequent incident DM, stratified by sex.

Physical activity participation	Men		Women	
	HR (95%CI)[a]	P value	HR (95%CI)	P value
**Global physical activity trajectories**				
Persistently low	Reference		Reference	
Initially low then improving	0.44 (0.30, 0.64)	<0.001	0.40 (0.27, 0.57)	<0.001
Initially high then declining	0.99 (0.50, 1.97)	0.979	0.60 (0.31, 1.16)	0.131
Persistently moderate	0.93 (0.65, 1.34)	0.703	0.57 (0.42, 0.77)	<0.001
Persistently high	0.73 (0.49, 1.09)	0.121	0.31 (0.20, 0.47)	<0.001
**Cumulative weighted global physical activity participation *Z* score (SD × year)**				
Per 10 units increment	0.95 (0.93, 0.98)	<0.001	0.91 (0.89, 0.94)	<0.001

[a] Adjusted for age × time, sex, ethnicity and education, cohabitation status, mobility status, current smoking, alcohol consumption, depressive symptoms, overweight status, hypertension, prediabetes status, stroke, cardiovascular diseases, chronic lung diseases and cancer.

Supplemental Table 6. Association of long-term physical activity participation on subsequent obesity assessed by BMI and waist circumference, stratified by age.

Physical activity participation	BMI (kg/m^2)		Waist circumference (cm)	
	β (95% CI)[a]	P value	β (95% CI)	P value
**Aged < 65 years**				
**Global physical activity trajectories**				
Persistently low	Reference		Reference	
Initially low then improving	-0.990 (-1.371, -0.609)	<0.001	-3.027 (-3.950, -2.104)	<0.001
Initially high then declining	-0.044 (-1.090, 1.003)	0.935	-0.491 (-3.028, 2.047)	0.705
Persistently moderate	-0.585 (-1.041, -0.130)	0.012	-2.237 (-3.340, -1.134)	<0.001
Persistently high	-1.641 (-2.122, -1.160)	<0.001	-5.469 (-6.636, -4.302)	<0.001
**Cumulative weighted global physical activity participation *Z* score (SD × year)**				
Per 10 units increment	-0.122 (-0.152, -0.092)	<0.001	-0.380 (-0.453, -0.308)	<0.001
**Aged ≥ 65 years**				
**Global physical activity trajectories**				
Persistently low	Reference		Reference	
Initially low then improving	-1.504 (-2.408, -0.600)	0.001	-3.118 (-5.309, -0.927)	0.005
Initially high then declining	-0.172 (-1.030, 0.686)	0.695	-1.806 (-3.788, 0.177)	0.074
Persistently moderate	-1.229 (-1.746, -0.711)	<0.001	-3.414 (-4.638, -2.190)	<0.001

Persistently high	-1.359 (-1.958, -0.760)	<0.001	-3.973 (-5.398, -2.548)	<0.001
**Cumulative weighted global physical activity participation *Z* score (SD × year)**				
Per 10 units increment	-0.095 (-0.136, -0.054)	<0.001	-0.308 (-0.405, -0.211)	<0.001

[a] Adjusted covariates included sex, age, ethnicity, education, cohabitation status, mobility status, current smoking, alcohol consumption, depressive symptoms, overweight status, hypertension, prediabetes status, stroke, cardiovascular diseases, chronic lung diseases and cancer.

Supplemental Table 7. Association of long-term physical activity participation on subsequent incident DM, stratified by age.

Physical activity participation	Aged < 65 years		Aged ≥ 65 years	
	HR (95%CI)[a]	P value	HR (95%CI)	P value
**Global physical activity trajectories**				
Persistently low	Reference		Reference	
Initially low then improving	0.44 (0.33, 0.58)	<0.001	0.58 (0.27, 1.24)	0.158
Initially high then declining	0.90 (0.48, 1.69)	0.747	0.51 (0.25, 1.05)	0.068
Persistently moderate	0.81 (0.61, 1.08)	0.152	0.44 (0.29, 0.67)	<0.001
Persistently high	0.46 (0.32, 0.65)	<0.001	0.55 (0.34, 0.87)	0.010
**Cumulative weighted global physical activity participation *Z* score (SD × year)**				
Per 10 units increment	0.93 (0.91, 0.95)	<0.001	0.95 (0.92, 0.99)	0.008

[a] Adjusted for age × time, sex, ethnicity and education, cohabitation status, mobility status, current smoking, alcohol consumption, depressive symptoms, overweight status, hypertension, prediabetes status, stroke, cardiovascular diseases, chronic lung diseases and cancer.

Supplemental Table 8. Association of long-term physical activity participation on subsequent obesity assessed by BMI and waist circumference, stratified by BMI.

Physical activity participation	BMI (kg/m²)		Waist circumference (cm)	
	β (95% CI)[a]	P value	β (95% CI)	P value
**BMI < 25 kg/m²**				
**Global physical activity trajectories**				
Persistently low	Reference		Reference	
Initially low then improving	-1.075 (-1.439, -0.711)	<0.001	-3.162 (-4.049, -2.275)	<0.001
Initially high then declining	-0.300 (-1.183, 0.584)	0.506	-2.021 (-4.125, 0.083)	0.060
Persistently moderate	-0.555 (-0.987, -0.123)	0.012	-2.320 (-3.365, -1.275)	<0.001
Persistently high	-1.359 (-1.823, -0.894)	<0.001	-4.753 (-5.880, -3.627)	<0.001
**Cumulative weighted global physical activity participation *Z* score (SD × year)**				
Per 10 units increment	-0.119 (-0.148, -0.090)	<0.001	-0.371 (-0.442, -0.300)	<0.001
**BMI ≥ 25 kg/m²**				
**Global physical activity trajectories**				
Persistently low	Reference		Reference	
Initially low then improving	-0.779 (-2.317, 0.760)	0.321	-2.194 (-5.887, 1.500)	0.244
Initially high then declining	-0.122 (-1.162, 0.918)	0.818	-0.738 (-3.126, 1.651)	0.545
Persistently moderate	-1.137 (-1.748, -0.526)	<0.001	-2.462 (-3.889, -1.036)	<0.001

Persistently high	-1.784 (-2.458, -1.110)	<0.001	-4.600 (-6.183, -3.017)	<0.001
**Cumulative weighted global physical activity participation *Z* score (SD × year)**				
Per 10 units increment	-0.102 (-0.147, -0.057)	<0.001	-0.313 (-0.419, -0.207)	<0.001

[a] Adjusted covariates included sex, age, ethnicity, education, cohabitation status, mobility status, current smoking, alcohol consumption, depressive symptoms, overweight status, hypertension, prediabetes status, stroke, cardiovascular diseases, chronic lung diseases and cancer.

Supplemental Table 9. Association of long-term physical activity participation on subsequent incident DM, stratified by BMI.

Physical activity participation	BMI < 25 kg/m^2		BMI ≥ 25 kg/m^2	
	HR (95%CI)[a]	P value	HR (95%CI)	P value
**Global physical activity trajectories**				
Persistently low	Reference		Reference	
Initially low then improving	0.42 (0.32, 0.55)	<0.001	0.39 (0.12, 1.29)	0.123
Initially high then declining	0.92 (0.51, 1.64)	0.775	0.52 (0.23, 1.17)	0.116
Persistently moderate	0.69 (0.51, 0.94)	0.018	0.62 (0.41, 0.94)	0.023
Persistently high	0.51 (0.36, 0.74)	<0.001	0.39 (0.24, 0.63)	<0.001
**Cumulative weighted global physical activity participation *Z* score (SD × year)**				
Per 10 units increment	0.93 (0.91, 0.95)	<0.001	0.93 (0.90, 0.96)	<0.001

[a] Adjusted for age × time, sex, ethnicity and education, cohabitation status, mobility status, current smoking, alcohol consumption, depressive symptoms, overweight status, hypertension, prediabetes status, stroke, cardiovascular diseases, chronic lung diseases and cancer.

Supplemental Table 10. Association of long-term physical activity participation on subsequent obesity assessed by BMI and waist circumference, after excluding participants reported any difficulties in activities of daily living during waves 1 to 4, or developed incident DM within two years after wave 4.

Physical activity participation	BMI (kg/m^2)		Waist circumference (cm)	
	β (95% CI)[a]	P value	β (95% CI)	P value
**Global physical activity trajectories**				
Persistently low	Reference		Reference	
Initially low then improving	-1.127 (-1.476, -0.778)	<0.001	-3.275 (-4.128, -2.422)	<0.001
Initially high then declining	-0.332 (-1.082, 0.417)	0.385	-2.103 (-3.902, -0.304)	0.022
Persistently moderate	-0.782 (-1.157, -0.408)	<0.001	-2.657 (-3.568, -1.745)	<0.001
Persistently high	-1.554 (-1.946, -1.162)	<0.001	-5.094 (-6.050, -4.137)	<0.001
**Cumulative weighted global physical activity participation *Z* score (SD × year)**				
Quintile 1	Reference		Reference	
Quintile 2	-0.862 (-1.252, -0.473)	<0.001	-2.907 (-3.857, -1.957)	<0.001
Quintile 3	-0.976 (-1.394, -0.557)	<0.001	-2.908 (-3.929, -1.886)	<0.001
Quintile 4	-1.408 (-1.816, -1.000)	<0.001	-4.389 (-5.384, -3.394)	<0.001
Quintile 5	-1.924 (-2.357, -1.491)	<0.001	-6.026 (-7.083, -4.969)	<0.001
Test for linear trend	-0.450 (-0.551, -0.349)	<0.001	-1.403 (-1.649, -1.156)	<0.001
Per 10 units increment	-0.112 (-0.137, -0.087)	<0.001	-0.361 (-0.421, -0.300)	<0.001

[a] Adjusted covariates included sex, age, ethnicity, education, cohabitation status, current smoking, alcohol consumption, depressive symptoms, overweight status, hypertension, prediabetes status, stroke, cardiovascular diseases, chronic lung diseases and cancer.

Supplemental Table 11. Association of long-term physical activity participation on subsequent incident DM, after excluding participants reported any difficulties in activities of daily living during waves 1 to 4, or developed incident DM within two years after wave 4.

Physical activity participation	Events/Total	Model 1[a]		Model 2[b]	
		HR (95% CI)	P value	HR (95% CI)	P value
**Global physical activity trajectories**					
Persistently low	204/2316	Reference		Reference	
Initially low then improving	78/1817	0.35 (0.26, 0.46)	<0.001	0.39 (0.30, 0.52)	<0.001
Initially high then declining	15/242	1.29 (0.76, 2.20)	0.349	0.77 (0.45, 1.34)	0.359
Persistently moderate	147/2209	1.23 (0.96, 1.56)	0.096	0.72 (0.55, 0.94)	0.016
Persistently high	99/1956	0.78 (0.59, 1.04)	0.095	0.48 (0.36, 0.66)	<0.001
**Cumulative weighted global physical activity participation Z score (SD × year)**					
Quintile 1	138/1631	Reference		Reference	
Quintile 2	97/1570	0.63 (0.47, 0.83)	<0.001	0.53 (0.40, 0.71)	<0.001
Quintile 3	116/1666	0.99 (0.76, 1.29)	0.935	0.50 (0.37, 0.68)	<0.001
Quintile 4	95/1803	0.64 (0.49, 0.85)	0.002	0.37 (0.27, 0.50)	<0.001
Quintile 5	97/1870	0.67 (0.49, 0.92)	0.013	0.32 (0.23, 0.45)	<0.001
Test for linear trend	-	0.91 (0.85, 0.98)	0.013	0.76 (0.70, 0.82)	<0.001
Per 10 units increment	-	0.98 (0.96, 1.00)	0.019	0.94 (0.92, 0.96)	<0.001

[a] Adjusted for age × time, sex, ethnicity and education.
[b] Additionally adjusted for cohabitation status, current smoking, alcohol consumption, depressive symptoms, overweight status, hypertension, prediabetes status, stroke, cardiovascular diseases, chronic lung diseases and cancer.

Supplemental Table 12. Association of long-term physical activity participation on subsequent incident DM recorded in 2 to 6 years after wave 4.

Physical activity participation	Incident DM in 2 years [a]		Incident DM in 4 years [a]		Incident DM in 6 years [a]	
	HR (95% CI)	P value	HR (95% CI)	P value	HR (95% CI)	P value
**Global physical activity trajectories**						
Persistently low	Reference		Reference		Reference	
Initially low then improving	1.40 (0.48, 4.09)	0.535	0.42 (0.25, 0.70)	<0.001	0.46 (0.30, 0.72)	<0.001
Initially high then declining	1.01 (0.23, 4.37)	0.990	0.71 (0.33, 1.53)	0.382	0.68 (0.36, 1.28)	0.236
Persistently moderate	0.22 (0.05, 0.90)	0.035	0.50 (0.30, 0.83)	0.007	0.65 (0.44, 0.97)	0.035
Persistently high	0.43 (0.11, 1.63)	0.216	0.85 (0.51, 1.44)	0.552	0.83 (0.53, 1.29)	0.399
**Cumulative weighted global physical activity participation Z score (SD × year)**						
Quintile 1	Reference		Reference			
Quintile 2	1.19 (0.47, 3.03)	0.714	0.56 (0.36, 0.88)	0.013	0.59 (0.40, 0.86)	0.007
Quintile 3	0.22 (0.05, 0.90)	0.035	0.38 (0.22, 0.64)	<0.001	0.52 (0.34, 0.78)	0.002
Quintile 4	0.23 (0.05, 1.08)	0.063	0.32 (0.18, 0.57)	<0.001	0.45 (0.28, 0.71)	<0.001
Quintile 5	0.21 (0.04, 1.06)	0.059	0.55 (0.31, 0.98)	0.044	0.57 (0.34, 0.93)	0.025
Test for linear trend	0.64 (0.44, 0.93)	0.018	0.81 (0.70, 0.94)	0.006	0.85 (0.75, 0.96)	0.008
Per 10 units increment	0.91 (0.83, 1.01)	0.073	0.96 (0.93, 1.00)	0.070	0.97 (0.93, 1.00)	0.037

[a] Adjusted for age × time, sex, ethnicity and education, cohabitation status, mobility status, current smoking, alcohol consumption, depressive symptoms, overweight status, hypertension, prediabetes status, stroke, cardiovascular diseases, chronic lung diseases and cancer.

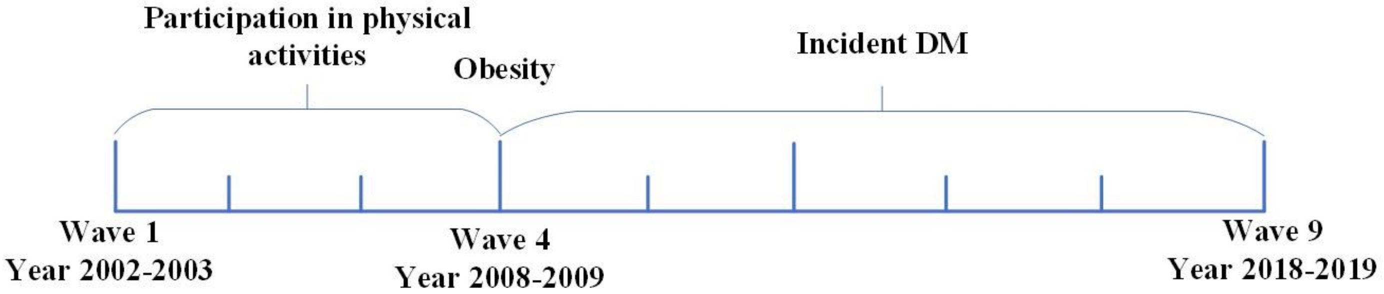

Supplemental Figure 1. Study timeline and design.

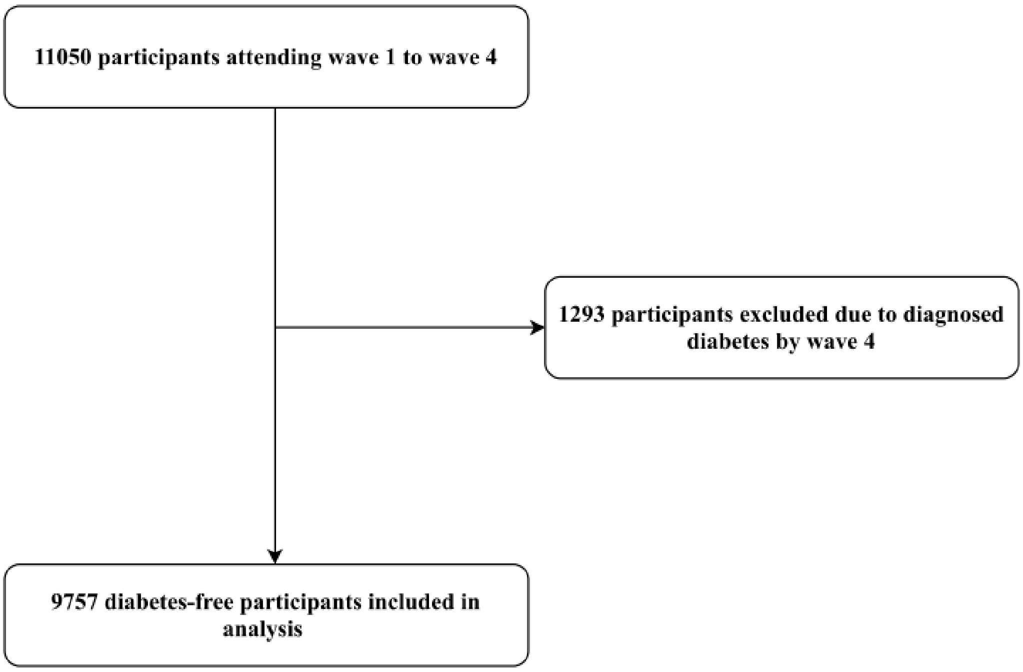

Supplemental Figure 2. Flow chart of participants selection.

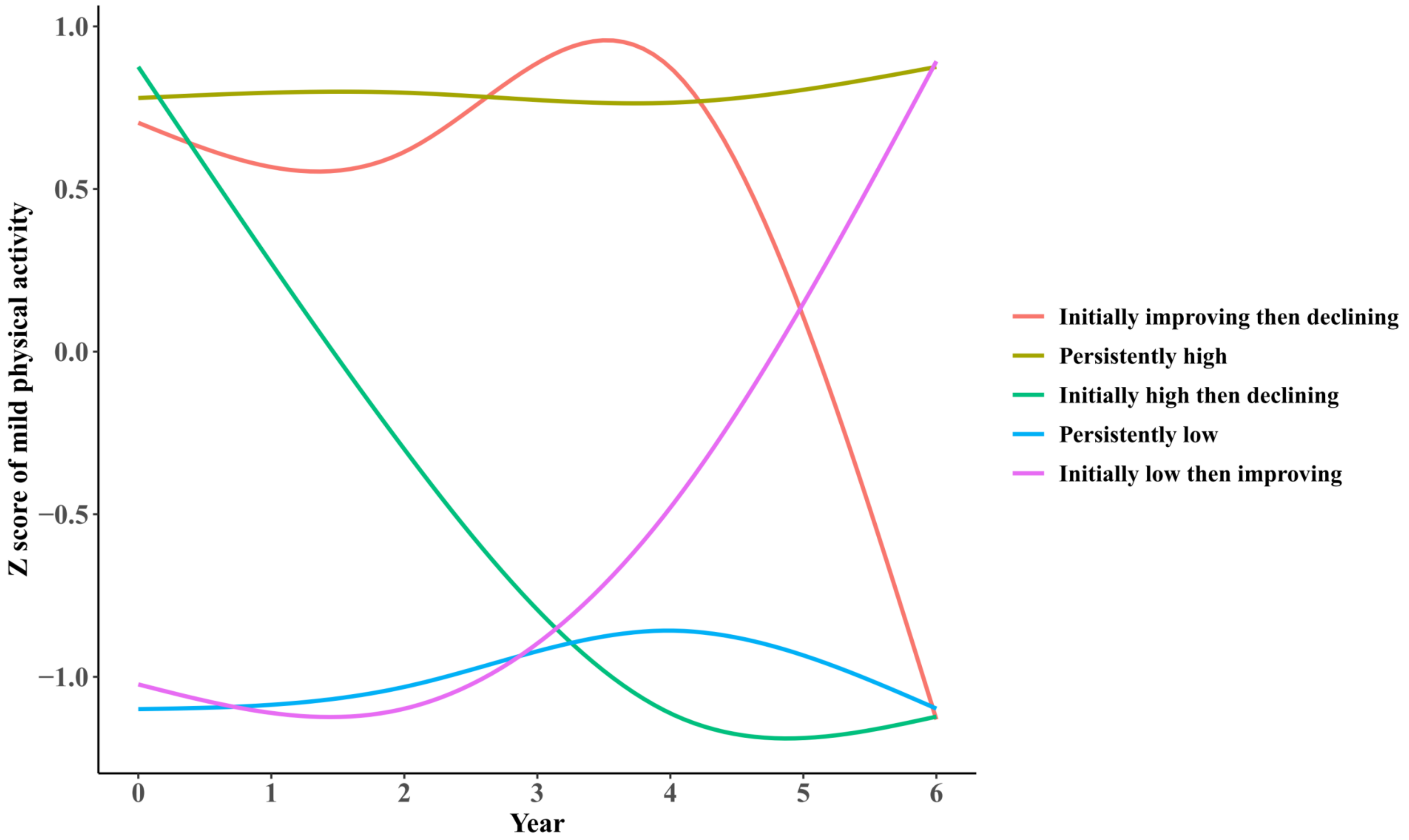
1.0
0.5
0.0
−0.5
−1.0
Z score of mild physical activity
0
1
2
3
4
5
6
Year
Initially improving then declining
Persistently high
Initially high then declining
Persistently low
Initially low then improving

Supplemental Figure 3. Trajectories of participation in mild intensity physical activities by participants from the ELSA over a 6-year span.

Identified trajectories included: 1) persistently low (N=577); 2) initially low then improving (N=3539); 3) initially high then declining (N=228); 4) persistently high (N=5092); 5) initially improving then declining (N=321).

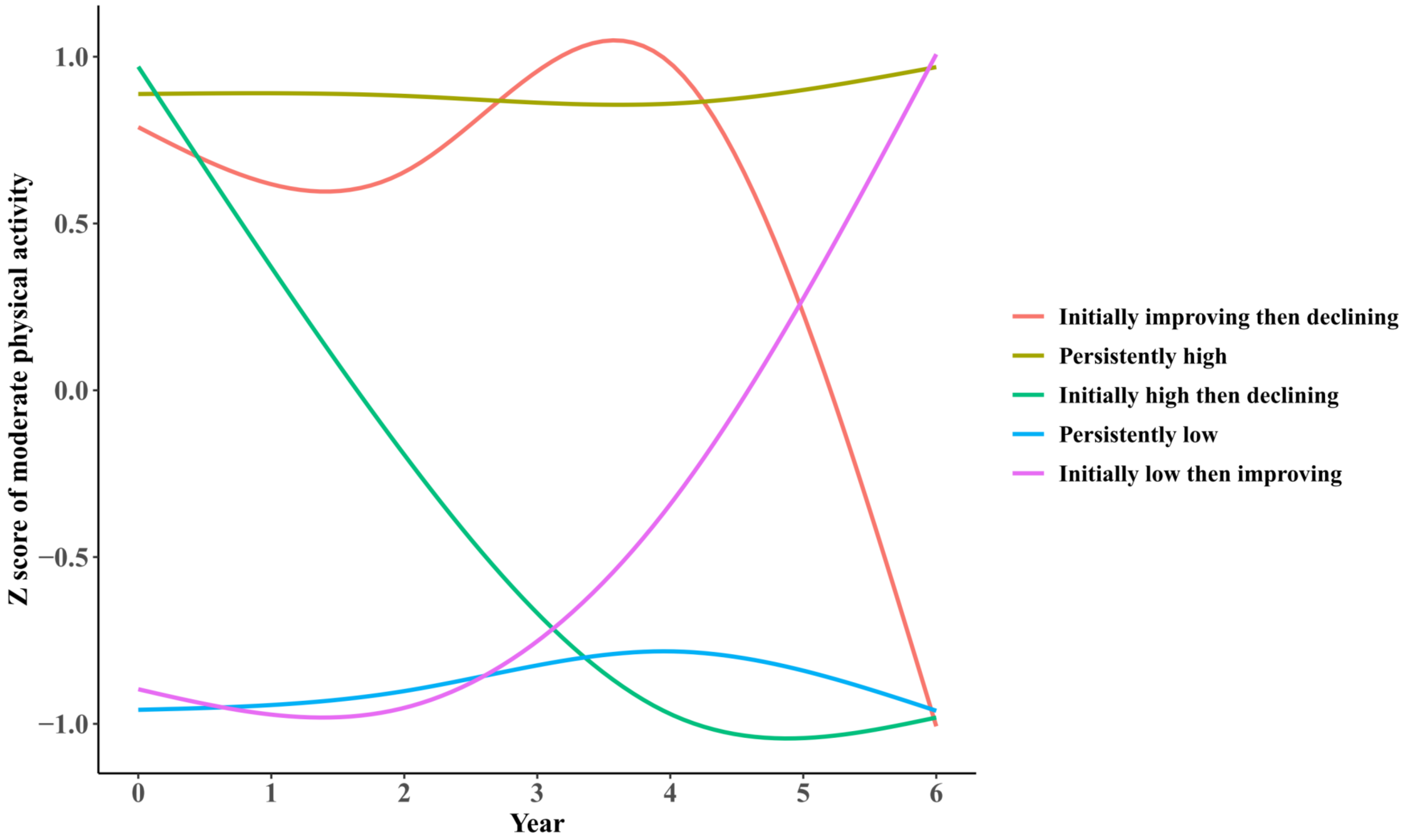

Z score of moderate physical activity
1.0
0.5
0.0
−0.5
−1.0
0
1
2
3
4
5
6
Year
Initially improving then declining
Persistently high
Initially high then declining
Persistently low
Initially low then improving

Supplemental Figure 4. Trajectories of participation in moderate intensity physical activities by participants from the ELSA over a 6-year span.

Identified trajectories included: 1) persistently low (N=1147); 2) initially low then improving (N=3333); 3) initially high then declining (N=385); 4) persistently high (N=4478); 5) initially improving then declining (N=414).

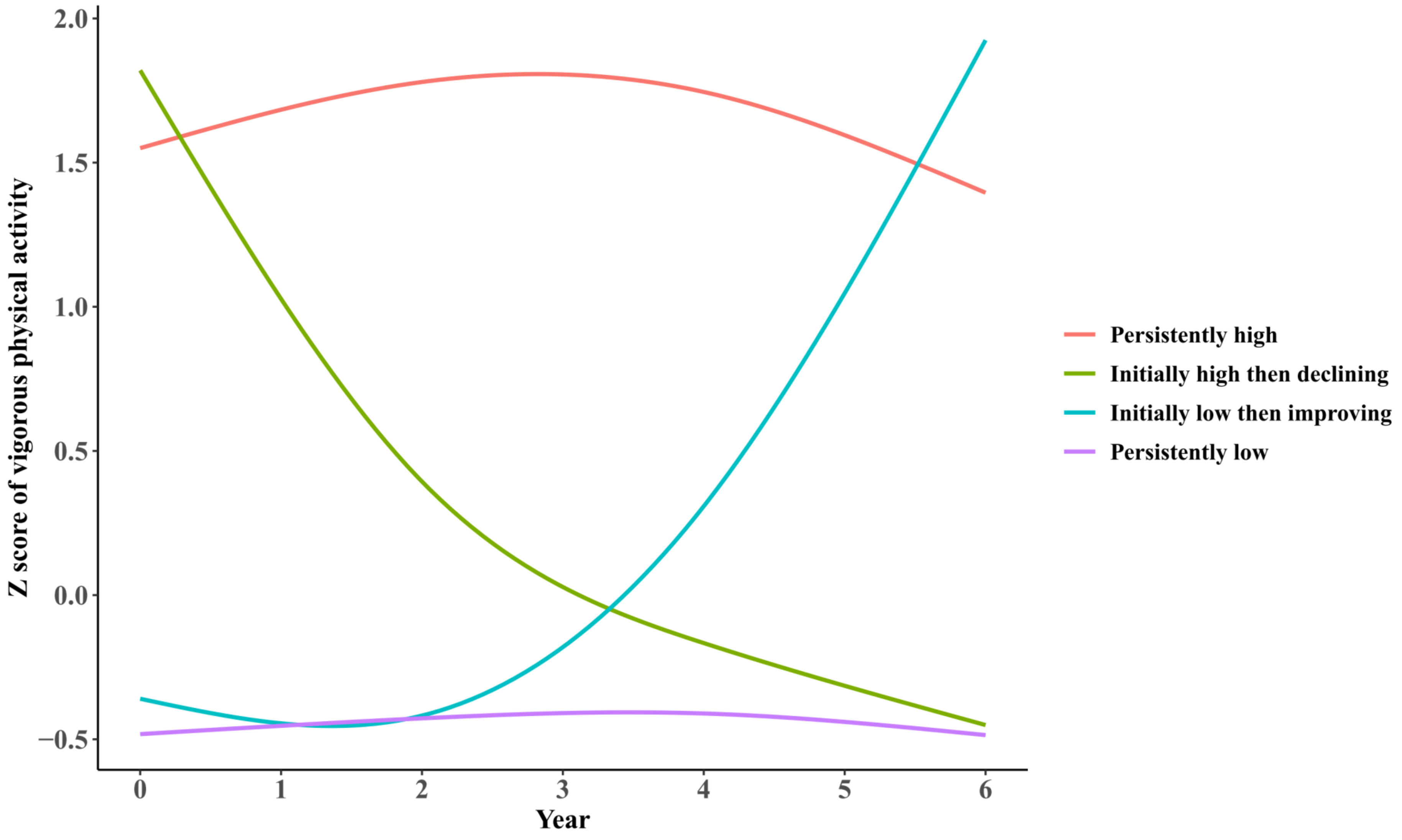
Z score of vigorous physical activity
2.0
1.5
1.0
0.5
0.0
−0.5
0
1
2
3
4
5
6
Year
Persistently high
Initially high then declining
Initially low then improving
Persistently low

Supplemental Figure 5. Trajectories of participation in vigorous intensity physical activities by participants from the ELSA over a 6-year span.

Identified trajectories included: 1) persistently low (N=5311); 2) initially low then improving (N=1973); 3) initially high then declining (N=894); 4) persistently high (N=1579).
